# rAAV-PHP.B escapes the mouse eye and causes lethality whereas rAAV9 can transduce aniridic corneal limbal stem cells without lethality

**DOI:** 10.1038/s41434-023-00400-6

**Published:** 2023-04-19

**Authors:** Seyedeh Zeinab Mirjalili Mohanna, Andrea J. Korecki, Elizabeth M. Simpson

**Affiliations:** 1 Centre for Molecular Medicine and Therapeutics at British Columbia Children’s Hospital, The University of British Columbia, Vancouver, BC Canada; 2 Department of Medical Genetics, The University of British Columbia, Vancouver, BC Canada

**Keywords:** Genetic transduction, Gene therapy, Visual system

## Abstract

Recently safety concerns have been raised in connection with high doses of recombinant adeno-associated viruses (rAAV). Therefore, we undertook a series of experiments to test viral capsid (rAAV9 and rAAV-PHP.B), dose, and route of administration (intrastromal, intravitreal, and intravenous) focused on aniridia, a congenital blindness that currently has no cure. The success of gene therapy for aniridia may depend on the presence of functional limbal stem cells (LSCs) in the damaged aniridic corneas and whether rAAV can transduce them. Both these concerns were unknown, and thus were also addressed by our studies. For the first time, we report ataxia and lethality after intravitreal or intrastromal rAAV-PHP.B virus injections. We demonstrated virus escape from the eye and transduction of non-ocular tissues by rAAV9 and rAAV-PHP.B capsids. We have also shown that intrastromal and intravitreal delivery of rAAV9 can transduce functional LSCs, as well as all four PAX6-expressing retinal cell types in aniridic eye, respectively. Overall, lack of adverse events and successful transduction of LSCs and retinal cells makes it clear that rAAV9 is the capsid of choice for future aniridia gene therapy. Our finding of rAAV lethality after intraocular injections will be impactful for other researchers developing rAAV-based gene therapies.

## Introduction

Gene therapy is undergoing a successful revival for disorders with unmet therapeutic needs. To date, recombinant adeno-associated viruses (rAAVs) are one of the most frequently used vectors for in vivo delivery of gene therapies [1]. rAAV-based gene therapies have shown acceptable long-term safety and efficacy profiles in several clinical trials, leading to FDA-approval of Luxturna in 2017 and Zolgensma in 2019 [2]. However, recent studies have raised safety concerns associated with high doses of rAAV in both preclinical studies [3–5] and clinical gene therapy trials [6, 7]. Therefore, when developing a new rAAV gene therapy, elements such as viral capsid, route of administration, dose, and promoter need to be carefully considered. Among these elements, we will explore the choice of capsid and route of administration in our study to pave the way for developing a new rAAV-based therapy for the eye disorder aniridia.

Aniridia is a rare congenital eye disorder, which is caused by autosomal dominant variants in the paired box 6 (*PAX6*) gene. It clinically manifests as reduced visual acuity and photophobia in childhood due to hypoplasia of the retinal fovea and iris, and gradually leads to blindness by the second to third decade of life due to glaucoma and/or slowly progressing corneal vascularization and clouding [8, 9]. This time course means there is a therapeutic window available to undertake treatment. To date, various symptomatic interventions are available for aniridia patients to slow the disease progress, but none prevent the eventual blindness. Limbal stem cell (LSC) deficiency has been proposed as the underlying mechanism responsible for corneal clouding in aniridia [10–12] and as an important contributing factor to the corneal epithelial thinning observed in this disease [13–16]. In a healthy cornea, the slow-dividing LSCs are present throughout life and continuously replenish the epithelium with new cells to maintain the transparency of the cornea [17]. This makes LSCs of the cornea important targets for intervention in patients with aniridia.

The rAAV capsid controls tropism and together with the route of administration has the ability to greatly influence the safety and efficacy of gene therapy [18]. To date, 13 naturally occurring AAV serotypes have been identified, and numerous variants within these serotypes have been isolated from various species including humans and non-human primates (NHPs) [19]. rAAVs were generated on the foundation of these naturally occurring capsids and have been studied by many groups to determine their tissue tropism [20]. rAAV9 is among the most-commonly used capsids for systemic delivery to the central nervous system (CNS), owning to its natural ability to cross the blood-brain barrier (BBB) after intravenous injection leading to neuronal tropism in neonate pups, and astrocytic tropism in adult mice [21] as well as in NHPs [22]. In the last decade, it has become increasingly popular to apply capsid engineering approaches such as directed evolution to further improve the transduction efficiency of rAAVs. In 2016, capsid engineering studies led to the development of rAAV-PHP.B (hereafter referred to as PHP.B) with superior BBB-crossing ability after intravenous injection in adult mice compared to its parental rAAV9 capsid [23]. The following year, a directed evolution strategy led to the discovery of a second-generation capsid, rAAV-PHP.eB, with further enhanced CNS transduction efficiency after systemic delivery in adult mice [24], creating new opportunities for capsid selection when developing gene therapy. However, shortly after, researchers discovered that the excellent properties of PHP.B capsids for systemic delivery to the CNS depends on interaction with the LY6A receptor and therefore are mouse strain [4, 25–27] and species-specific [4, 28, 29]. In spite of this, the PHP.B family of capsids is still widely used in preclinical gene therapy studies [30–35] and are valuable in demonstrating efficacy in a mouse model prior to NHP studies, recognizing that the final capsid selection must be optimal for humans.

Intravitreal injections of rAAV9 have previously proven effective for the transduction of various PAX6-expressing retinal cells in wild-type (WT) mouse eyes [36–38]. These studies used a “current” marker (e.g., green fluorescent protein) showing where the virus is currently, which may be suitable for modeling traditional gene-augmentation therapies. In contrast, much less work has been done with rAAV carrying a “historical” marker (e.g., Cre, rearranging the genome), which aims to show all cells transduced over time and thus is better suited for modeling newer gene editing therapies such as CRISPR. Cre has been previously used in the cornea to show the ability of intrastromal injection of rAAV8[Y733F] to reach PAX6-expressing corneal epithelial cells, as well as the therapeutically significant corneal LSCs in WT mouse eyes [39]. Now it is important to build on these works and explore the transduction patterns of rAAV9 and the novel PHP.B capsids in the aniridic retina and cornea using a historical marker to make the findings translatable, especially to gene editing strategies for this disease.

There is a good mouse model of aniridia known as Small eye (*Sey*), which is also caused by a dominant variant in the *Pax6* gene [16, 40]. The corneal abnormalities seen in these mice mimic those of aniridia patients [16] and have been similarly attributed to defects in LSCs [41]. The *Sey* mice used in this study have been extensively characterized [14] and bred to additionally carry a loxP-3xStop-loxP-tdTomato Cre-reporter allele [42]. The resulting mice have previously been used for testing and optimizing gene editing delivery methods for aniridia [43]. Injecting these reporter mice with rAAV expressing improved Cre (iCre) removes the transcriptional stop signal upstream of tdTomato gene, resulting in tdTomato epifluorescence expression that can be visualized at the single cell level in histological analysis, allowing characterization of vector target-cell specificity. This has been demonstrated for corneal LSCs in WT mice when transduction of LSCs led to tdTomato expression, the cells divided and gave rise to tdTomato positive transient amplifying cells, that became terminally differentiated epithelial cells of the central cornea [39]. This cell movement in the corneal epithelial layer generated a red stripe-like banding pattern and indicated the successful targeting of functional LSCs [39].

Here we present our findings from testing two capsids (rAAV9 and PHP.B), in two mouse strains (WT and *Sey*), by three different administration routes (intrastromal, intravitreal, and intravenous). We recommend the best capsid and administration routes for future gene therapy for aniridia. In addition, these findings deliver an important message of rAAV lethality for all rAAV ocular gene therapies in development.

## Materials and methods

### Mouse husbandry and breeding

Experimental mice were primarily B6129F1-loxP-3xStop-loxP-tdTomato, *Pax6*^*Sey/+*^ or B6129F1-loxP-3xStop-loxP-tdTomato, *Pax6*^*+/+*^, of both sexes, produced as the first generation by crossing homozygous B6.Cg-*Gt(ROSA)26Sor*^*tm14(CAG-tdTomato)Hze*^/J (JAX strain: 007914, aka Ai14) dams to 129S1/SvImJ-*Pax6*^*Sey/+*^ (MMRRC Stock No: 050624-MU) studs. A subset of experimental mice was B6129F1-loxP-3xStop-loxP-tdTomato, Hmgcr-lacZ, *Pax6*^*Sey/+*^ or B6129F1-loxP-3xStop-loxP-tdTomato, Hmgcr-lacZ, *Pax6*^*+/+*^, of both sexes, produced by a two-generation breeding strategy. Initially, homozygous B6-loxP-3xStop-loxP-tdTomato dams were crossed to B6-Hmgcr-lacZ (JAX strain: 024050) studs. The resulting homozygous B6-loxP-3xStop-loxP-tdTomato, Hmgcr-lacZ dams were then crossed to 129S1/SvImJ-*Pax6*^*Sey/+*^ studs. (Note: Hmgcr-lacZ was not used in these experiments.) The non-reporter control mice (i.e., without the loxP-3xStop-loxP-tdTomato, or Hmgcr-lacZ alleles) were either B6129F1-*Pax6*^*Sey/+*^ or B6129F1-*Pax6*^*+/+*^, of both sexes, produced as the first generation by crossing C57BL/6J (JAX strain: 000664) dams to 129S1/SvImJ-*Pax6*^*Sey/+*^ or 129S1/SvImJ (JAX strain: 002448) studs.

Mice were maintained in the pathogen-free Centre for Molecular Medicine and Therapeutics (CMMT) mouse facility on a 7 a.m.–7 p.m. light cycle, 20 ± 2 °C with 50 ± 5% relative humidity, and had food and water ad libitum. All procedures involving mice were in accordance with the Canadian Council on Animal Care and UBC Animal Care Committee (Protocols A17-0204, A17-0205, A21-0140, and A21-0184).

Pups were weaned and ear notched at postnatal day (P) 21. Collected ear notches were digested in 200 µl of tissue homogenization buffer with Proteinase K (Cat #P2308, Sigma-Aldrich, Burlington, MA) according to a previously described protocol [44]. PCR-based genotyping was carried out to determine the presence of the *Sey* variant or Hmgcr-lacZ using Taq DNA Polymerase (Cat #18038042, Invitrogen, Waltham, MA). Primer pair oEMS6071 (5′-TCACTCTATTTTCCCAACACAGCC-3′) and oEMS6073 (5′-CTGAGCTTCATCCGAGTCTTCTTA-3′) were used for identifying *Sey* mice. Primer pair oEMS6087 (5′-AAAGCAGGATTTATGGCAAGGTG-3′) and oEMS6088 (5′-AGTTTGAGGGGACGACGACAGTA-3′) was used for identifying Hmgcr-lacZ-positive mice.

### rAAV production

Small chicken beta actin promoter/CMV enhancer (smCBA) was ordered for direct DNA synthesis (GenScript, Piscataway, NJ), and cloned into the multiple cloning site (*Avr*II, *Fse*I, *Mlu*I, and *Asc*I) of our “plug and play” rAAV2 backbone plasmid (pEMS1987) [45]. This genome plasmid includes: an intron (optimized chimeric; 173 bp) (Promega, Madison, MI) [46], *Not*I flanked iCre recombinase (1032 bp) [47], *Asi*SI flanked woodchuck hepatitis virus post-transcriptional regulatory element (WPRE) mut6 (587 bp) [48], and SV40 polyA (222 bp) (Promega) sequences. The resulting plasmid, pEMS2118 (Addgene plasmid #49143), was propagated in *E.coli* SURE cells (Cat #200227, Agilent Technologies, Santa Clara, CA). DNA was prepared by QIAGEN Spin MiniPrep Kit (Cat #27104, QIAGEN, Germantown, MD) and plasmid was confirmed free of rearrangements by *Ahd*I digest, inverted terminal repeats verified by *Sma*I digest, and cloning sites verified by Sanger sequencing. Confirmed plasmid was then sent to the University of Pennsylvania Vector Core (Philadelphia, PA) for large-scale DNA amplification using the EndoFree Plasmid Mega Kit (Cat #12381, QIAGEN, Hilden, Germany). Quality control on the plasmid preparation was done by *Sma*I, *Pvu*II, and *Sna*BI digests, and the confirmed plasmid was then packaged into either rAAV9 or PHP.B capsid by the University of Pennsylvania Vector Core. The rAAV genome titer was determined by droplet digital PCR using primers that recognize inverted terminal repeats.

### Intravenous injections

Sample sizes (number of mice per group) were based on a previously published similar experiment [45]. Pups were randomly grouped based on the litter and all were injected regardless of eye phenotype. P21 injections were into the tail vein. A 31-gauge needle on a 0.33 cc syringe (Cat #320440, BD, Franklin Lakes, NJ) was inserted into the tail vein and 50 µl of virus at a titer of either 1 × 10^13^, 3 × 10^12^, 1.5 × 10^12^, 6 × 10^11^, or 3 × 10^11^ viral genome/ml (vg/ml) of PHP.B ssAAV-smCBA-iCre-WPRE (hereafter referred to as PHP.B iCre) in phosphate buffer saline (PBS) and 0.001% Pluronic acid (Cat #24040032, Gibco, Thermo Fisher, Waltham, MA) (together, PBS + P) was slowly injected. Final injection doses were: 5 × 10^11^ (16 mice), 1.5 × 10^11^ (16 mice), 7.5 × 10^10^ (7 mice), 3 × 10^10^ (7 mice), and 1.5 × 10^10^ (7 mice) vg/mouse. Mice were observed daily for 5 days following injection, followed by weekly observations.

### Intravitreal and intrastromal injections

Sample sizes (number of mice per group) were based on a previously published similar experiment [45]. Mice were genotyped and grouped by one person and injections were done on a separate day by a different person who did not re-examine the eyes. Adult mice (2 months old for intravitreal injections and 3 months old for intrastromal injections) were weighed, anesthetized with isoflurane inhalation, and injected with 5 mg/kg Metacam (DIN: 02240463) for pain management. All procedures were performed while mice were at surgical plane of anesthesia. Alcaine eye drops (DIN: 00035076) were applied to numb the eye receiving the injection, and Optixcare (Aventix, Burlington, Canada) lubricating eye gel was applied to the contralateral eye to prevent dryness. Custom-made 33- and 34-gauge removable Small Hub RN needles (length: 9.53 mm; point style: 4; bevel angle: 30°) (Hamilton Company, Reno, NV) were used for intravitreal and intrastromal injections, respectively. For rAAV injections, 2 μl of undiluted virus at a titre of either 4.2 × 10^13^ vg/ml of PHP.B iCre or 3.8 × 10^13^ vg/ml of rAAV9 ssAAV-smCBA-iCre-WPRE (hereafter referred to as rAAV9 iCre) in PBS + P was loaded into a 2.5 μl syringe (Cat #7632-01, Hamilton Company). For vehicle-only injections, 2 μl of PBS + P was loaded into the syringe. Final injection doses were: 8.4 × 10^10^ vg/mouse for PHP.B iCre and 7.6 × 10^10^ vg/mouse for rAAV9 iCre. Intravitreal injection was performed by inserting the needle perpendicular to the sclera close to the limbus region and slowly advancing within sclera to vitreous chamber before dispensing the virus. Intrastromal injection was performed following a previously described method [39, 43]. Briefly, the needle was inserted into the stroma near the corneoscleral junction and was carefully advanced to the center of the stroma where the payload was dispensed. The central injection method in combination with the injection volume were chosen with the goal of reaching as much of the cornea as possible after a single injection. Injection success was a predetermined inclusion criterion for intrastromal injections, as this is a challenging technique to perform on the small mouse eye. Mice that did not receive a successful intrastromal injection were immediately euthanized. Mice were weighed and eyes closely examined daily for five days following injection. After that, mice were monitored weekly until the first lethality was observed, leading to careful examination of all the remaining mice twice a week until they were euthanized and tissues were harvested from a subset.

### Slit lamp imaging

Slit lamp imaging was performed according to a previously described method [49]. Briefly, 3-month-old mice were placed under general anesthesia. The Phoenix Micron Anterior Segment Imaging system (Phoenix Research Labs, Pleasanton, CA) was used to visualize the corneal phenotype of the mouse left eye.

### Histology

Intraocularly-injected mice were initially planned to be harvested at 1 month and 7 months post-injection, but due to the unforeseen lethality in PHP.B iCre mice, the experimental mice were euthanized and tissues were harvested once they reached a humane endpoint and the non-reporter control mice were euthanized without harvesting. Despite the absence of phenotype in rAAV9 iCre mice, harvest time for these mice was also adjusted to match the PHP.B iCre mice for comparison purposes. However, in the case of intrastromal rAAV9 iCre injections, a subset of injected mice was aged and harvested at 7 months post-injection to allow for the formation of LSC stripes. At the time of harvest, mice were euthanized by anesthetic avertin (Cat #T48402, Sigma-Aldrich) overdose, and transcardially perfused according to a previously described method [50]. Tissues were then harvested and post-fixed overnight in 4% paraformaldehyde in PBS at 4 °C. On the following day, for cryosections, tissues were transferred to 25% sucrose in PBS for a minimum of 24 h at 4 °C or until they were processed. Tissues were embedded in Tissue-Tek optimal cutting temperature compound (Cat #4583; Sakura FineTek, Torrance, CA) in cryomolds on dry ice. Next, 20 μm thick cryosections were cut and directly mounted onto Superfrost Plus slides (Cat #1255015, Thermo Fisher). Slides were stored at −80 °C until staining was performed. For staining, slides were warmed to room temperature for 5 min and pressed underweight for 30–60 min. Cryosections were then washed twice in PBS and incubated with Hoechst nuclear stain (1:1000) in PBS for 10 min. Following incubation, cryosections were washed twice more with PBS, before being mounted with ProLong Gold Antifade reagent (Cat #P10144, Invitrogen). Slides were kept in dark at 4 °C until visualization. For corneal flat mounts, fixed eyes were stored in PBS until they were processed. Eyes were placed in a petri dish filled with PBS. Corneas were dissected using surgical scissors (Cat #15000-03, Fine Science Tools, Foster City, CA) and forceps (Cat #11251-33, Fine Science Tools) under a Leica MZ APO dissecting microscope (Leica Microsystems, Wetzlar, DE). Four radial cuts were made into the corneas before transferring them onto slides (one cornea per slide). Corneas were then mounted with ProLong Gold Antifade reagent with DAPI (Cat #P36935, Invitrogen) and flattened with a cover slip. Slides were kept in dark at 4 °C overnight before imaging. All injected eyes were processed and analyzed, whereas non-ocular tissues from a minimum of 2 mice per genotype were processed and analyzed for each group at each harvest time point. Results were confirmed by an individual blinded to treatment groups.

### Microscopy and image processing

tdTomato epifluorescence was visualized in cryosections and corneal flat mounts using an Olympus BX61 fluorescent microscope (Olympus, Tokyo, Japan) at either 4× magnification (cerebellum, spinal cord, liver, heart, and pancreas) or 10× magnification (eye cryosections and corneal flat mounts). The cerebellum and eye images were taken as Tile scans. Corneal flat mounts were re-imaged as Tile scans using a Leica SP8 confocal microscope (Leica Microsystems) at 20× magnification by z-projection through the epithelium that was defined at the center of each cornea. The resulting images were processed using Image J (http://rsbweb.nih.gov/ij/), and Illustrator (Adobe, Mountain View, CA). In some images, Hoechst/DAPI was enhanced consistently throughout the entire image for better visualization.

### Statistical analysis

GraphPad Prism version 9.1.1 for Windows (GraphPad Software, San Diego, CA, www.graphpad.com) was used for all statistical analysis and plotting Kaplan–Meier survival curves with 95% confidence intervals (based on the asymmetrical method) [51]. Log-rank test was performed for statistical comparison of the survival curves and *p* < 0.05 was accepted as statistically significant.

## Results

### PHP.B iCre injections caused lethality in mice, regardless of administration route

Our previous work with rAAV9 shows its favorable tropism for the retina and cornea after intraocular injections in WT mice [36–38]. However, rAAV9 is known to be inefficient in transducing the retina after intravenous injection in adult mice [52]. At the time this study began, PHP.B was chosen as the most advanced capsid for non-invasive systemic delivery to the retina in adult mice [23]. Therefore, the PHP.B capsid was used for all three administration routes (intravenous, intravitreal, and intrastromal), whereas the use of rAAV9 was limited to intraocular injections.

**Fig. 1:**
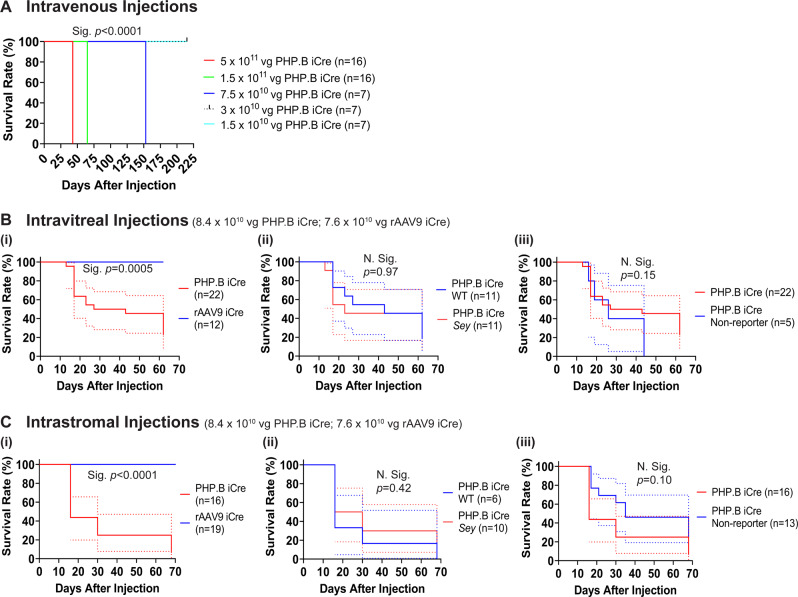
Mice injected with PHP.B or rAAV9 iCre viruses had different survival curves. Cages were closely examined for phenotype once or twice a week. Animals were euthanized once they reached a humane endpoint and tissues were harvested from a subset. Kaplan-Meier survival curves (solid or dashed lines) were generated with 95% confidence intervals (dotted lines). **A** Intravenous injections of high doses of PHP.B iCre (5 × 10^11^, 1.5 × 10^11^, and 7.5 × 10^10^ vg) caused severe adverse events leading to euthanasia of the entire dose group on the same day. Intravenous injections of high doses of PHP.B iCre significantly reduced the probability of survival compared to the groups that received lower doses (3 × 10^10^ and 1.5 × 10^10^ vg). **B(i)**, **C(i)** Intravitreal and intrastromal injections of PHP.B iCre significantly reduced the probability of survival compared to rAAV9 iCre injected mice. **B(ii)**, **C(ii)** The probability of survival was not significantly different between the PHP.B iCre injected WT and *Sey* mice. **B(iii)**, **C(iii)** The probability of survival was not significantly different between the PHP.B iCre injected tdTomato reporter and non-reporter mice. Log-rank test was used to determine the significance of the data. iCre improved Cre, N Sig non-significant, *Sey* *Pax6* small eye, Sig significant, vg viral genome, WT wild type.

Intravenous injections were carried out at five doses of PHP.B iCre virus, with mice injected at the three highest doses showing a progressive phenotype leading to lethality (Fig. 1A). Uninjected mice served as controls. Mice injected with the highest dose of 5 × 10^11^ vg/mouse were first observed to have a mild “clasping” phenotype for their hind legs 33 days post-injection. The clasping phenotype progressed to hind leg paralysis and these animals were euthanized 43 days post-injection. Mice injected with the second highest dose of 1.5 × 10^11^ vg/mouse were first observed to have a mild clasping phenotype for their hind legs 58 days post-injection, with the phenotype progressing quickly, the remaining mice were euthanized 65 days post-injection. Mice injected with the third highest dose of 7.5 × 10^10^ vg/mouse were first observed to have a very mild clasping phenotype in their hind legs at 69 days post-injection, with the phenotype progressing to severe and mice beginning to have difficulty moving around the cage due to hind leg paralysis at 153 days post-injection, at which point the mice were euthanized. Mice injected with the fourth highest dose (second lowest) of 3 × 10^10^ vg/mouse showed a mild clasping phenotype seven months (215 days) post-injection. Mice injected with the lowest dose of 1.5 × 10^10^ vg/mouse showed no phenotype at seven months (215 days) post-injection, which was the experimental end point. The Kaplan-Meier survival curves were significantly different (*p* < 0.0001) among the five groups according to the log-rank test. Although the lowest intravenous dose (1.5 × 10^10^ vg/mouse) did not result in any observable phenotype, we concluded that this dose was probably too low to have a therapeutic impact. Therefore, to continue to compare both capsids we chose to focus on intraocular (intravitreal or intrastromal) injections.

To maximize transduction, and because we did not anticipate any deleterious impact of intraocular injections, undiluted PHP.B and rAAV9 iCre viruses were used for intravitreal injections. Throughout these experiments, non-reporter virus injected mice and vehicle-only injected mice served as controls to account for the potential impact of transgene expression or delivery method on the mice wellbeing. Experimental mice and non-reporter control mice were intravitreally injected with 8.4 × 10^10^ vg/mouse of PHP.B iCre. Surprisingly, we found a dead mouse among the experimental mice 13 days post-injection. Upon careful examination of the remaining injected animals, mice in both the experimental and non-reporter control groups, displayed ataxia and weight loss and were subsequently harvested between 17 and 62 days post-injection when they reached a humane endpoint. The ataxia phenotype observed was characterized by motor coordination and balance deficits, as well as head and body tremors. Overall, this phenotype had a more rapid onset, was faster progressing, and more severe than what we observed after the intravenous injections. It is worth noting that ataxia is not part of the aniridia phenotype in either patients with heterozygous *PAX6* loss-of-function variants [53] or *Sey* mice [14]. Importantly, in a parallel study, experimental mice and non-reporter control mice were intravitreally injected with 7.6 × 10^10^ vg/mouse of, not PHP.B, but rAAV9 iCre. At the time of harvest (62 days post-injection), these mice did not display any adverse events. As expected, no phenotype was observed in mice that received vehicle-only injections.

Similar to intravitreal injections, to maximize transduction, PHP.B and rAAV9 viruses were used at their highest possible dose for intrastromal injections. These injections were initiated at the time when we did not anticipate any deleterious impact of intrastromal injection. Non-reporter virus injected mice and vehicle-only injected mice served as controls. Experimental mice and non-reporter control mice were intrastromally injected with 8.4 ×10^10^ vg/mouse of PHP.B iCre. Surprisingly, a dead mouse was observed at 16 days post-injection. Except for one mouse in the experimental group, all remaining mice in both experimental and non-reporter control injected groups displayed varying degrees of ataxia and weight loss after the injections and were subsequently harvested between 16-68 days post-injection when they reached a humane endpoint. The fast-progressing ataxia in these mice was similar to our observations after intravitreal injections. Importantly, in a parallel study, experimental mice and non-reporter control mice were intrastromally injected with 7.6 ×10^10^ vg/mouse of, not PHP.B, but rAAV9 iCre. At the time of harvest (68 days post-injection), these mice did not display any adverse events. As expected, no phenotype was observed in mice that received vehicle-only injections.

Kaplan-Meier survival analysis showed significantly reduced survival in mice intraocularly injected (intravitreal or intrastromal) with PHP.B iCre compared to those injected with rAAV9 iCre (log-rank test, *p* = 0.0005 for intravitreal injections and *p* < 0.0001 for intrastromal injections) (Fig. 1Bi and Ci). Survival curves were not significantly different between WT and *Sey* mice that received intraocular injections of PHP.B iCre (log-rank test, *p* = 0.97 for intravitreal injections and *p* = 0.42 for intrastromal injections) (Fig. 1Bii and Cii). This demonstrates that survival of these mice was not influenced by *Pax6* genotype. Lastly, survival curves were not significantly different between experimental and non-reporter control groups that received intraocular PHP.B iCre injections (log-rank test, *p* = 0.15 for intravitreal injections and *p* = 0.10 for intrastromal injections) (Fig. 1Biii and Ciii). Hence, the observed lethality was attributed to the PHP.B iCre virus, rather than tdTomato or lacZ expression.

### Intraocular injected rAAVs escaped from the eye

**Fig. 2:**
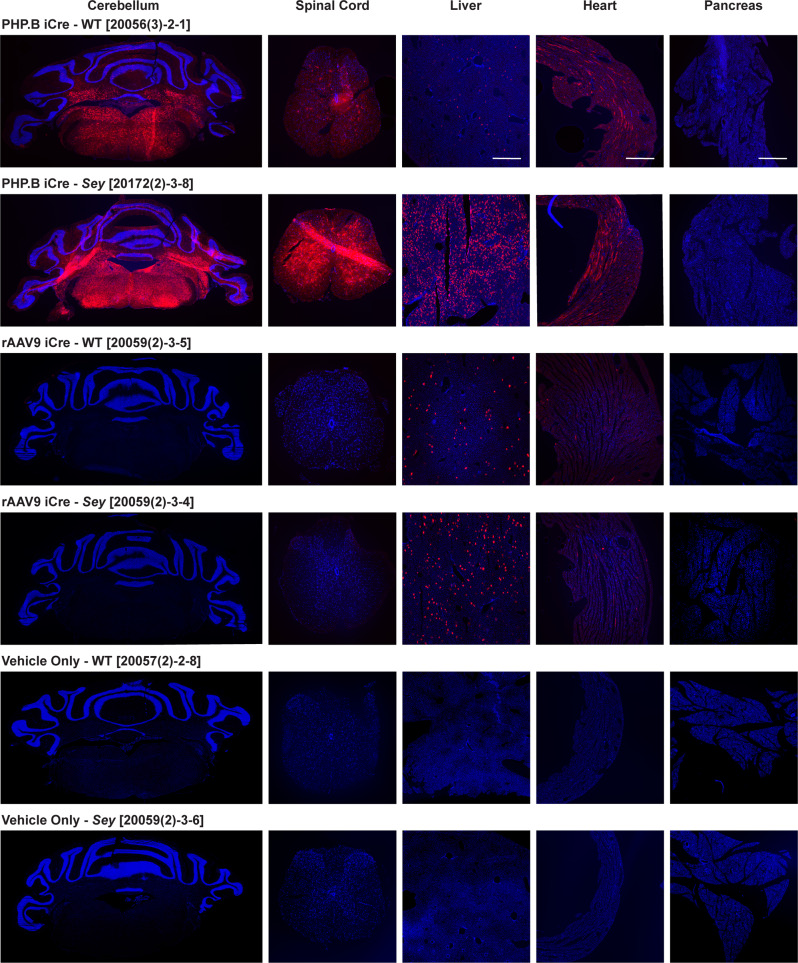
Intravitreally-delivered PHP.B and rAAV9 iCre viruses escaped the eye. Fluorescence images of mouse tissues (cerebellum, spinal cord, liver, heart, and pancreas). Injection doses were: 8.4 × 10^10^ vg/mouse for PHP.B iCre and 7.6 × 10^10^ vg/mouse for rAAV9 iCre. Mice were perfused 17–30 days after intravitreal injection of iCre viruses (see Methods for more detail). Tissues from a minimum of 2 mice per genotype were analyzed. The sectioned tissues were evaluated for tdTomato epifluorescence. The tdTomato signal was observed in the central nervous system cerebellum, and spinal cord of PHP.B iCre injected WT and *Sey* mice, but not in rAAV9 iCre injected mice. In contrast, tdTomato signal was detected in the peripheral tissues liver and heart, but was almost absent in pancreas, in both PHP.B iCre and rAAV9 iCre injected WT and *Sey* mice. tdTomato expression was not observed in age-matched vehicle-only injected mice (negative controls). The number in brackets refers to the mouse ID. Scale bar = 500 μm. Blue Hoechst nuclear stain, iCre improved Cre, red tdTomato epifluorescence, *Sey* *Pax6* small eye, WT wild type.

**Fig. 3:**
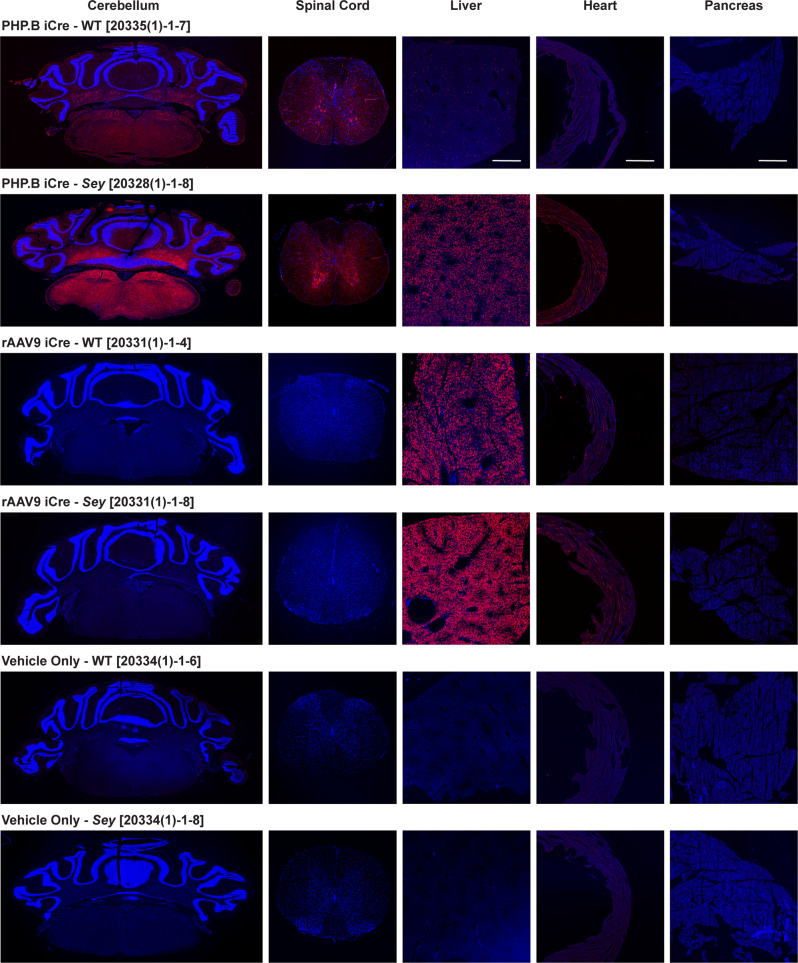
Intrastromally-delivered PHP.B and rAAV9 iCre viruses escaped the eye. Fluorescence images of mouse tissues (cerebellum, spinal cord, liver, heart, and pancreas). Injection doses were: 8.4 × 10^10^ vg/mouse for PHP.B iCre and 7.6 × 10^10^ vg/mouse for rAAV9 iCre. Mice were perfused 16–30 days after the intrastromal injection of iCre viruses (see Methods for more detail). Tissues from a minimum of 2 mice per genotype were analyzed. The sectioned tissues were evaluated for tdTomato epifluorescence. The tdTomato signal was observed in the central nervous system cerebellum, and spinal cord of PHP.B iCre injected WT and *Sey* mice, but not in rAAV9 iCre injected mice. In contrast, tdTomato signal was detected in the peripheral tissues liver and heart, but was almost absent in pancreas, in both PHP.B iCre and rAAV9 iCre injected WT and *Sey* mice. tdTomato expression was not observed in age-matched vehicle-only injected mice (negative controls). The number in brackets refers to the mouse ID. Scale bar = 500 μm. Blue Hoechst nuclear stain, iCre improved Cre, red tdTomato epifluorescence, *Sey* *Pax6* small eye, WT wild type.

To determine the rAAV transduction in non-target cells after intraocular injections (intravitreal or intrastromal), we evaluated cerebellum, spinal cord, liver, heart, and pancreas for the presence of tdTomato expression (Figs. 2 and 3). Histological analysis of early-harvested (16–30 days post-injection) tissues revealed tdTomato expression in the liver and heart, but was almost absent in pancreas, in both PHP.B and rAAV9 iCre injected WT and *Sey* mice. This demonstrates the ability of both these capsids to escape from the eye, regardless of the route of administration. Nevertheless, no tdTomato expression was observed in the cerebellum and spinal cord of mice intraocularly injected with rAAV9 iCre, which were the mice that did not display any adverse events. Whereas the PHP.B iCre injected mice demonstrated strong tdTomato expression in these tissues, regardless of the route of administration, and demonstrated severe ataxia and lethality. Histological analysis of tissues from vehicle-only control mice did not show any tdTomato expression.

### In PHP.B ocular-injected mice tdTomato signal directly correlated with severity of ataxia

**Fig. 4:**
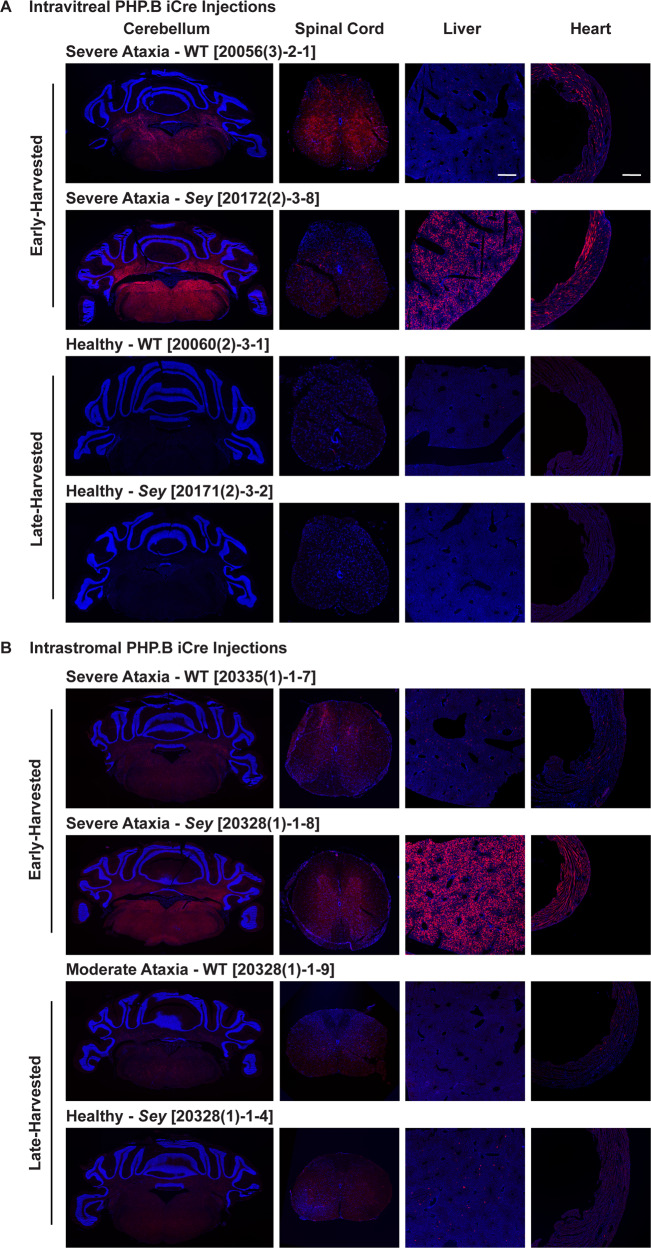
In PHP.B ocular-injected mice tdTomato signal was higher in ataxic versus healthy mice. Fluorescence images of tissues from early-harvested mice (17 days post-intravitreal injection and 16 days post-intrastromal injection) with severe ataxia compared to late-harvested mice (62 days post-intravitreal injection and 68 days post-intrastromal injection). **A** Intravitreal PHP.B iCre injections: Tissues from WT and *Sey* mice with severe ataxia exhibited a higher tdTomato intensity and spread in comparison to healthy mice. **B** Intrastromal PHP.B iCre injections: Tissues from WT and *Sey* mice with severe ataxia exhibited a higher tdTomato intensity and spread in comparison to healthier mice. The number in brackets refers to the mouse ID. A subset of mice presented in Figs. 2 and 3 was re-analyzed for this comparison. Scale bar = 500 μm. Blue Hoechst nuclear stain, iCre improved Cre, red tdTomato epifluorescence, *Sey* *Pax6* small eye, WT wild type.

We compared tissues from early-harvested (16-17 days post-injection) mice that had severe ataxia with late-harvested (62–68 days post-injection) mice to better understand the correlation between tdTomato expression and the severity of phenotype. For the intravitreal PHP.B iCre injections of experimental mice, we had two healthy WT and three healthy *Sey* mice at the end of the study. For the intrastromal injections we only had one healthy *Sey* mouse at the end of the study, as the only WT mouse that made it to 68 days had moderate ataxia, it was included in this analysis. In order to do a head-to-head comparison, tissues from early-harvested PHP.B iCre experimental mice (Figs. 2 and 3) were re-analyzed together with late-harvested tissues under the same histology and imaging conditions. We found that for both WT and *Sey* mice, tdTomato signal intensity and spread was higher in the cerebellum, spinal cord, liver, and heart of mice with severe ataxia compared to healthy mice after intravitreal PHP.B iCre injections (Fig. 4A). Similarly, for the intrastromal PHP.B iCre injections, tdTomato signal intensity and spread was higher in the cerebellum, spinal cord, liver, and heart of mice with severe ataxia compared to healthier mice (Fig. 4B).

### Intravitreal PHP.B and rAAV9 iCre injections resulted in transduction of a variety of retinal cells

**Fig. 5:**
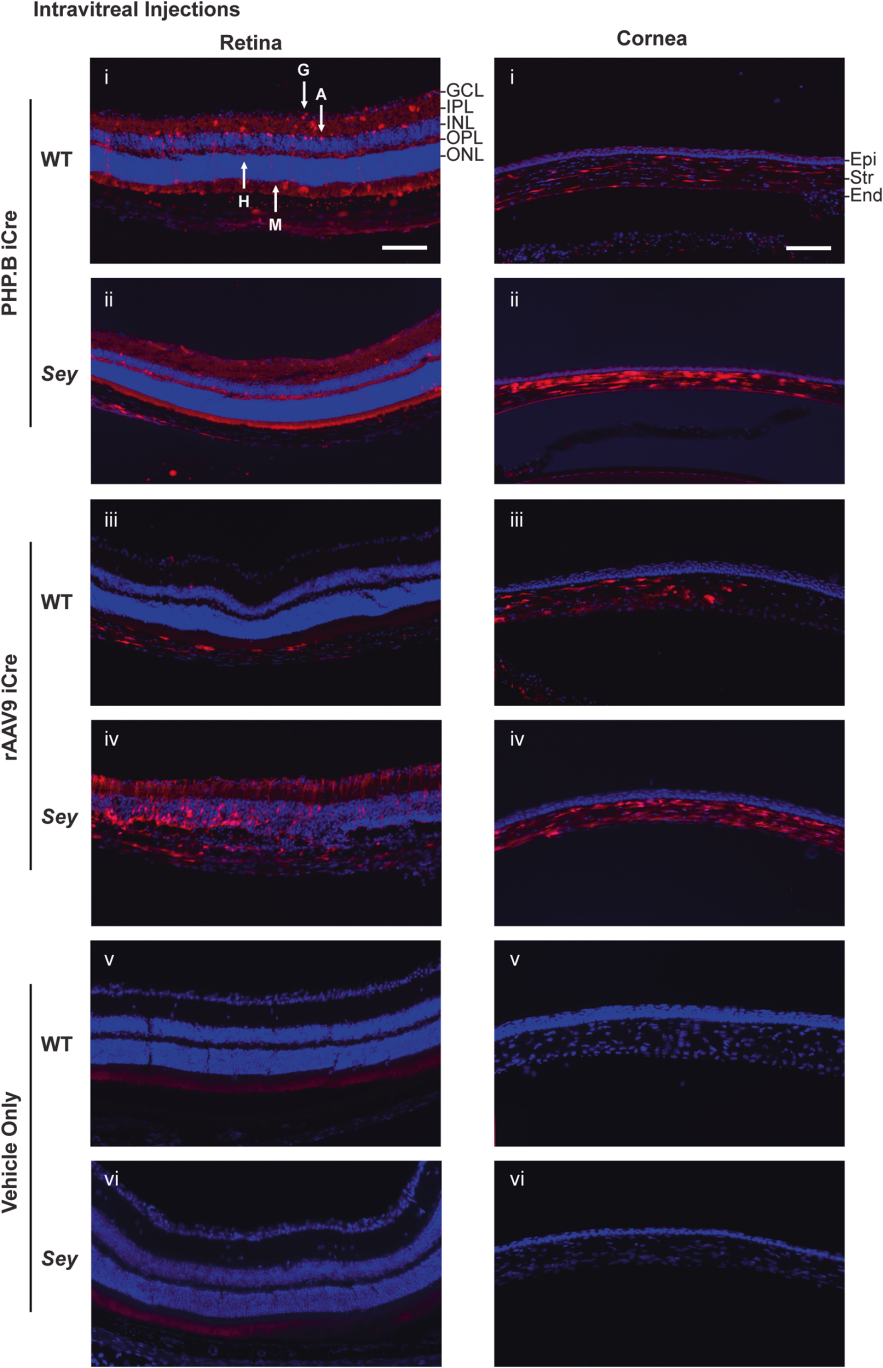
Intravitreally-delivered iCre viruses successfully transduced a variety of retinal cells and some corneal cells. Fluorescence images of injected mouse retinas and corneas. Injection doses were: 8.4 × 10^10^ vg/mouse for PHP.B iCre and 7.6 × 10^10^ vg/mouse for rAAV9 iCre. Intravitreal injection of PHP.B iCre into WT mice resulted in tdTomato expression in all four PAX6-expressing cells of the retina, ganglion, amacrine, horizontal, and Müller glia. rAAV9 iCre injection resulted in tdTomato expression in three PAX6-expressing cells, excluding Müller glia. But, intravitreal injections of PHP.B and rAAV9 iCre into *Sey* mice resulted in the expression of tdTomato in all PAX6-expressing cells of the retina. Intravitreal injections of PHP.B and rAAV9 iCre viruses also resulted in tdTomato expression in the stromal and endothelial layers of the cornea, but tdTomato expression was almost absent from the epithelial layer in both WT and *Sey* mice. tdTomato expression was not observed in age-matched vehicle-only injected eyes (negative controls). Retinal cells were identified by their location and morphology. Images with the same Roman numeral belong to one mouse, and correspond to overview images in Fig. S1. For the PHP.B iCre WT and *Sey* groups, each fluorescence image is representative of 11 eyes. For the rAAV9 iCre WT and *Sey* groups, each fluorescence image is representative of 4 and 8 eyes, respectively. Scale bar = 100 μm. A amacrine, blue Hoechst nuclear stain, End endothelium, Epi epithelium, G ganglion, GCL ganglion cell layer, H horizontal, iCre improved Cre, INL inner nuclear layer, IPL inner plexiform layer, M Müller glia, ONL outer nuclear layer, OPL outer plexiform layer, red tdTomato epifluorescence, *Sey* *Pax6* small eye, Str stroma, WT wild type.

Gene therapy for aniridia will be focused on delivering virus to the PAX6-expressing cells of the retina (ganglion, amacrine, horizontal, and Müller glia) and cornea (LSCs and epithelial). Intravitreal PHP.B iCre injections resulted in tdTomato expression in all four PAX6-expressing cells of the retina (identified by location and morphology), in both WT and *Sey* mice (Figs. 5 and S1). Whereas, intravitreal injection of rAAV9 iCre into WT mice led to tdTomato expression in all but the Müller glia. In contrast, in *Sey* mice tdTomato expression was observed in all four PAX6-expressing cells. Interestingly, tdTomato expression was not restricted to the retina despite the intravitreal injection site being close to the retina (Fig. S1). Expression was also observed in the corneal stromal and endothelial cells, but was almost absent from the epithelial layer in both WT and *Sey* mice, regardless of rAAV capsid. tdTomato expression was absent in the vehicle-only injected eyes (negative controls).

### Intrastromal PHP.B and rAAV9 iCre injections resulted in transduction of all three corneal cell layers

**Fig. 6:**
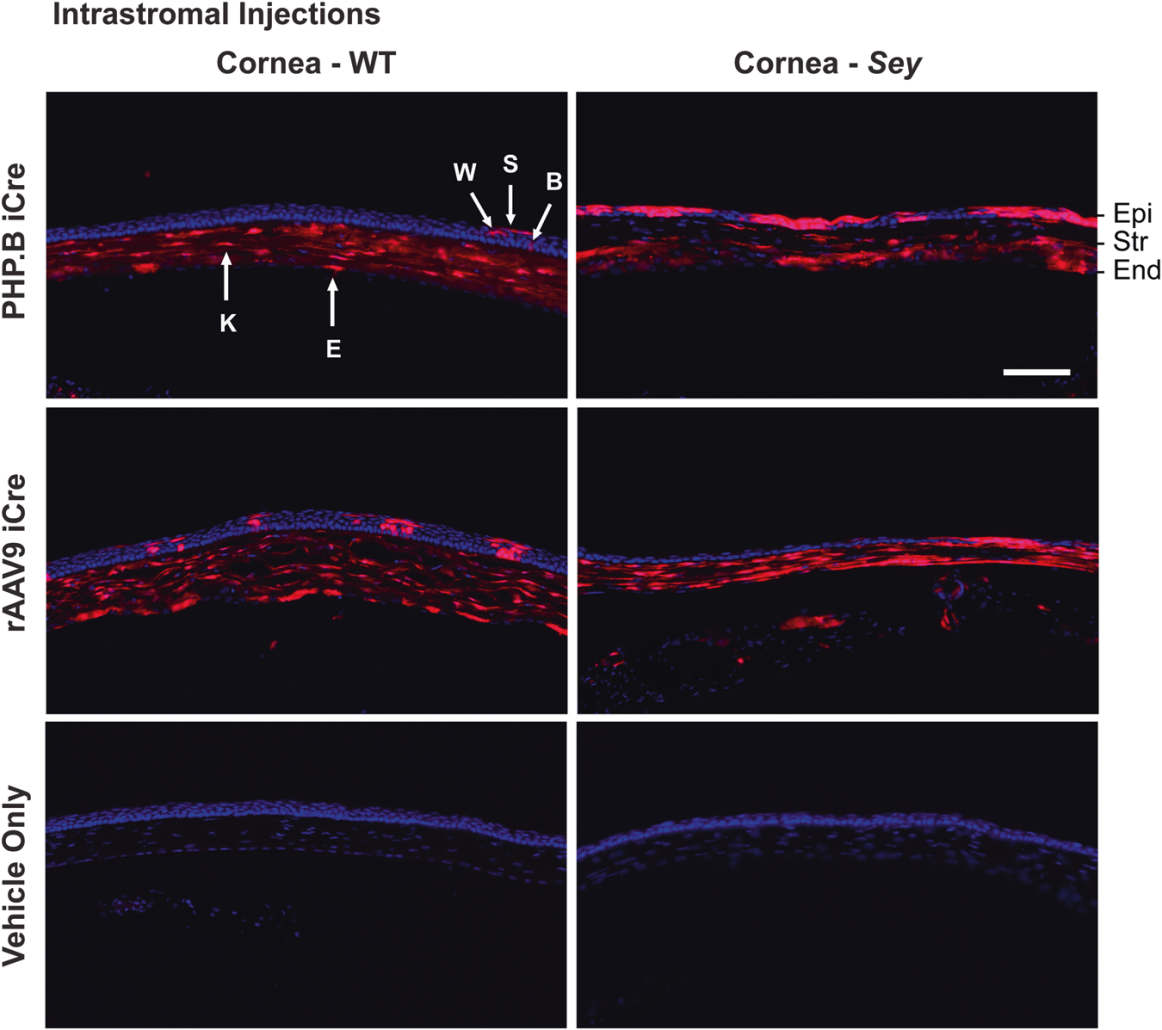
Intrastromally-delivered iCre viruses successfully transduced all three corneal cell layers. Fluorescence images of injected mouse corneas. Injection doses were: 8.4 × 10^10^ vg/mouse for PHP.B iCre and 7.6 × 10^10^ vg/mouse for rAAV9 iCre. Intrastromal injections of PHP.B and rAAV9 iCre showed successful transduction of cells in all three layers of the cornea (Epi, Str, and End) in WT and *Sey* mice. In the corneal epithelial layer, tdTomato expression was seen in basal cells, wing cells, and squamous cells. Sections were chosen to show all the transduced cell types in one image. Overall, the results did not show a bias in transduction of corneal cell types between PHP.B and rAAV9. tdTomato expression was not observed in age-matched vehicle-only injected eyes (negative controls). Corneal cells were identified by their location and morphology. Images with the same Roman numerals belong to one mouse, and correspond to overview images in Fig. S2. For the PHP.B iCre WT and *Sey* groups, each fluorescence image is representative of 6 and 10 eyes, respectively. For the rAAV9 iCre WT and *Sey* groups, each fluorescence image is representative of three eyes. Scale bar = 100 μm. B basal cell, blue Hoechst nuclear stain, E endothelial cell, End endothelium, Epi epithelium, iCre improved Cre, K keratocyte, red tdTomato epifluorescence, S squamous cell, *Sey* *Pax6* small eye, Str stroma, W wing cell, WT wild type.

Intrastromal PHP.B and rAAV9 iCre injections resulted in tdTomato expression in all three corneal cell layers, encompassing corneal epithelial cells (basal cells, wing cells, and squamous cells), stromal cells (keratocytes), and endothelial cells, identified by location and morphology in both WT and *Sey* mice (Fig. 6 and S2). Additionally, sectioning demonstrated that the central intrastromal injection method used resulted in even transduction throughout the cornea (Fig. S2). tdTomato was only expressed in the retinal blood vessels of intrastromally-injected mice (Fig. S2). tdTomato expression was absent in the vehicle-only injected eyes (negative controls).

### Intrastromal rAAV9 iCre injections successfully transduced corneal LSCs in adult aniridic mouse eyes

**Fig. 7:**
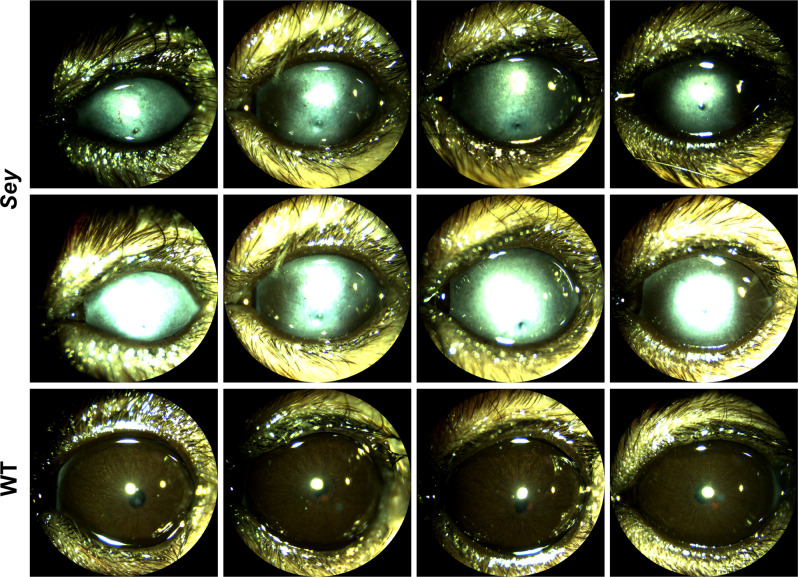
Corneal clouding was present in 3-month-old *Sey* corneas. Representative slit-lamp images of uninjected *Sey* and WT eyes at 3 months old. *Sey* eyes were imaged at two light settings to better capture the cloudiness of the corneas. All *Sey* eyes displayed severe corneal clouding, whereas age-matched WT eyes had transparent corneas. *Sey* *Pax6* small eye, WT wild type.

**Fig. 8:**
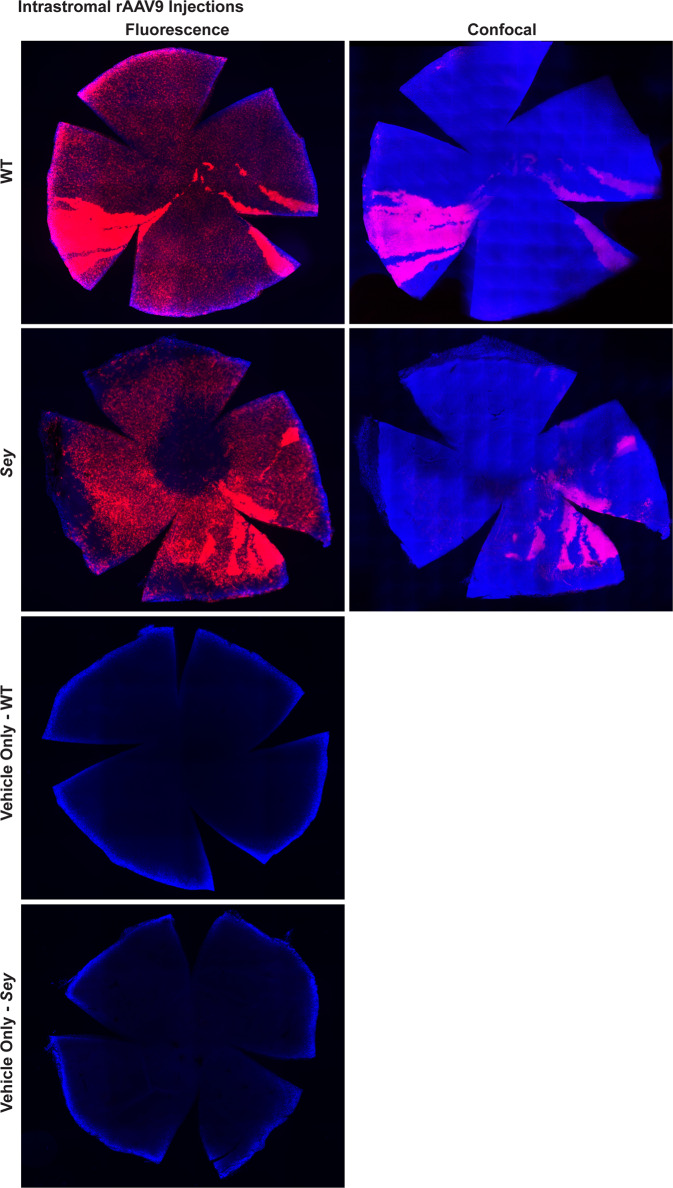
Intrastromal injections of rAAV9 iCre successfully transduced corneal LSCs in *Sey* mice. Representative fluorescence and confocal images of WT and *Sey* corneal flat mounts 7 months after injection. Corneal flat mounts were first examined under the fluorescence microscope to assess tdTomato expression in all three layers of the cornea, and then subjected to confocal microscopy to focus specifically on the entire corneal epithelial layer. Intrastromal injections of rAAV9 iCre resulted in tdTomato stripes indicative of LSC transduction in both WT and *Sey* corneas. tdTomato expression was not observed in age-matched vehicle-only injected corneal flat mounts (negative controls). Blue DAPI nuclear stain, red tdTomato epifluorescence, *Sey* *Pax6* small eye, WT wild type.

In *Sey* eyes, the exact age at which the LSC deficiency manifests is unknown. In this study, all intrastromal injections were performed when mice were 3 months old. By 3 months, the cloudy cornea phenotype of aniridia is already apparent in the *Sey* eyes (Fig. 7). The success of an rAAV gene therapy targeted towards LSC deficiency may depend on whether any functional LSCs are still present in the damaged *Sey* corneas at the time of injection. To investigate this, we looked at WT and *Sey* corneas 7 months after intrastromal rAAV9 iCre injections. It must be noted that tdTomato expression at 7 months post-injection is not a reflection of continuing virus presence, but is due to the permanent genomic rearrangement by the iCre/loxP recombination system that makes the transduced cells, and any cells descending from them, to persistently express tdTomato. Fluorescence microscopy demonstrated widespread tdTomato expression in the cornea of both WT and *Sey* mice (Fig. 8). Confocal microscopy confirmed that the tdTomato stripes are located in the corneal epithelium. In general, in the *Sey* eyes, the tdTomato stripes that originated from the limbus, as expected did not reach the center of the cornea [41]. Overall, two out of three WT corneas and five out of eight *Sey* corneas showed tdTomato stripes in the epithelium, indicating that functional LSCs are present in 3-month-old *Sey* corneas, which can be successfully transduced with rAAV9. tdTomato expression was absent in the cornea of vehicle-only injected mice (negative controls).

## Discussion

For ocular gene therapy, rAAV has been a popular choice [1]. Given the deteriorating state of the aniridic eye [54], a non-ocular delivery system such as intravenous was quite attractive. However, recent news of several deaths associated with intravenous delivery of high-dose rAAV therapies [6, 7] represents a major setback for the field and emphasizes the importance of establishing maximally-tolerated viral dose during preclinical gene therapy studies. Thus, the primary goal of our intravenous rAAV dose-response studies was to find the maximally-tolerated viral dose. Unfortunately, we concluded that the only dose of PHP.B iCre that did not cause toxicity in our mice was probably too low to be a viable option for our gene therapy. Thus, to continue to compare both capsids we shifted to the option of intraocular delivery.

Luxturna, an rAAV2-based augmentation gene therapy delivered intraocularly was approved in 2017 by the FDA for treating patients with a rare form of inherited vision loss [55]. Given this, and the intravenous toxicity, we shifted our focus to intraocular delivery with an aim to concentrate the rAAV in the eye and improve the safety and efficacy profiles. We were surprised to see severe ataxia and early lethality in our intravitreally-injected mice when the PHP.B capsid was used. It was even more shocking to see severe ataxia and early lethality after intrastromal PHP.B iCre injections, considering that the injection was done into the cornea, an avascular tissue in WT eyes. These observations were especially unexpected since in the literature ataxia has not been reported with the use of PHP.B in mice, and currently the only report of PHP.B-associated ataxia comes from the study in piglets that received high dose of intravenous injection of this virus [3]. Thus, this study is the first report of ataxia and lethality after intraocular injections of PHP.B. Furthermore, the adverse events that we observed in our study after intravitreal and intrastromal injections of PHP.B were substantially more severe than the local ocular toxicity (defined as morphological changes to the retina and retinal pigment epithelium) that another group observed in mice after subretinal injections of multiple rAAVs (although not PHP.B) [56]. Interestingly, the PHP.B capsid was used by an independent group for successful intravitreal delivery of CRISPR/Cas9 components to the retina of mouse pups (1 × 10^9^ vg/eye) [57]. The absence of toxicity could be due to lower virus dose, different promoter and regulatory elements, but is most likely due to the very short harvest time of 2 weeks. In our work, we are confident that the observed lethality is not due to promoter, viral genome dose, or vector preparation, as both PHP.B (lethal) and rAAV9 (non-lethal) iCre viruses had the exact same viral genome; were used at comparable doses; and were prepared in the same facility. Our findings are consistent with a group that showed intravenous injection of PHP.B virus at a high dose in an adult NHP resulted in severe acute toxicity and ultimately euthanasia, while the animal that received the same dose of rAAV9 showed no clinical symptoms [4]. Together, these results highlight the inherent differences in tropism in even closely related capsids (PHP.B and rAAV9) that can dramatically impact safety.

The eye is an easily accessible and contained structure, and therefore is widely considered to be a safe target for gene therapy [58]. Typically, tissue transduction after escape from the eye has not been reported, even for the PHP.B capsid [57]. However, several studies have performed biodistribution analysis after intraocular rAAV2 [59], rAAV8 [60–63], or rAAV8G9 [64] injections and detected vector genome in extraocular tissues. A limitation of these studies is that they do not make it clear whether or not extraocular cells had been transduced. In the current study, histological analysis demonstrated that both PHP.B and rAAV9 viruses escaped from the eye after intravitreal and intrastromal injections, and then transduced peripheral tissues. The “historical” reporter used in this study (a genomic rearrangement turning on tdTomato), may have more effectively captured the true extent of the viral escape since transduced cells are permanently and clearly marked. This is compared to the more commonly used “current” reporters (e.g., green fluorescent protein), for which signal is determined by viral copy number, and there is no signal if the virus is lost from the cell. Applying Occam’s razor to explain both intravitreal and intrastromal escapes, we suggest that PHP.B and rAAV9 capsids have crossed the adult blood-ocular barrier from the eye side into the bloodstream, resulting in extensive transduction of non-ocular tissues. In the future, we would recommend replacing a ubiquitous promoter as used here, with a target-cell-specific promoter to minimize transgene expression in off-target tissues [36–38, 45].

In this study, we must consider the mechanism whereby PHP.B and rAAV9, which both reached the bloodstream regardless of route of administration, differentially resulted in ataxia and death (PHP.B lethal and rAAV9 non-lethal). It is important to note that in adult mice, PHP.B capsids outperform rAAV9 in crossing the BBB and transducing the CNS [23, 25]. Our results support this in that we saw transduction of cerebellum and spinal cord only with the lethal PHP.B capsid. Another candidate region from NHP studies would be the dorsal root ganglia (DRG) [3, 5, 65], however, DRG are not protected by the BBB and both PHP.B and rAAV9 capsids have been shown in NHP to have similar tropism for DRG [4]. Thus, in our study, we conclude that the ataxia phenotype observed with PHP.B is most likely due to differential transduction of the cerebellum and/or spinal cord.

LSC deficiency has been proposed as the underlying mechanism responsible for corneal clouding, a major contributor to blindness in aniridia [10–12]. Thus, LSC are a critical therapeutic target. Intrastromal injection of rAAV8[Y733F] has been shown to transduce LSCs in adult WT mice [39], but similar data was not available for *Sey* mice. We know from other work that PAX6 haploinsufficiency results in fewer functional LSCs compared to WT corneas [41]. Nevertheless, the exact age of onset for LSC deficiency in the *Sey* cornea is unclear and therefore there was uncertainty whether rAAVs would be able to transduce these therapeutically important cells in an already cloudy *Sey* cornea. Here we have shown for the first time that 3-month-old cloudy *Sey* corneas still have functional LSCs and that they can be transduced with rAAV9. As expected, the tdTomato stripes from these LSCs fail to reach the center of the *Sey* corneas, consistent with the previously observed defective migration [41]. Regarding route of administration, we were surprised to see that intravitreal injection of rAAV9 was not only excellently suited for transducing the therapeutically important retinal cells, but it also reached the cornea. Since it would be exciting if one injection could transduce both the therapeutic retinal and corneal LSC targets, this observation deserves further study. Overall, our findings are most promising in the context of a future gene therapy for aniridia, which can transduce LSCs and may restore the normal migration pattern from these cells, thereby eliminating corneal clouding and the resulting blindness.

In this study, we conclude that PHP.B iCre can cause lethality in mice regardless of the administration route (intravenous, intravitreal, or intrastromal). Furthermore, we hypothesize the mechanism of escape from the eye by both capsids lies in their ability to cross adult blood-ocular barrier from the eye to the bloodstream, leading to the transduction of non-ocular tissues. Their differential tropism for CNS is the likely explanation for the observed lethality phenotype with PHP.B only. Most importantly, we have demonstrated that functional LSCs are present in the cloudy cornea of 3-month-old *Sey* mice. Lack of adverse events and successful transduction of the therapeutically important corneal LSCs and retinal cells with rAAV9 in the *Sey* eyes makes this capsid a good candidate for use in future aniridia gene therapy development. Finally, our findings regarding rAAV lethality will be impactful for other researchers working on developing rAAV-based gene therapies for ocular disease.

## Supplementary information

Supplementary Material

## Data Availability

The rAAV genome plasmid used in this study (pEMS2118) is available to the research community through the nonprofit distributor Addgene (plasmid #49143) (www.addgene.org).
