# Supplementary material for: rAAV-PHP.B escapes the mouse eye and causes lethality whereas rAAV9 can transduce aniridic corneal limbal stem cells without lethality
Source: Gene Ther. 2023 Apr 19;30(9):670–84. doi: 10.1038/s41434-023-00400-6 (PMC10506911; doi:10.1038/s41434-023-00400-6)
Supplement: Supplementary file 1 — Supplementary Material [file 41434_2023_400_MOESM1_ESM.pdf]

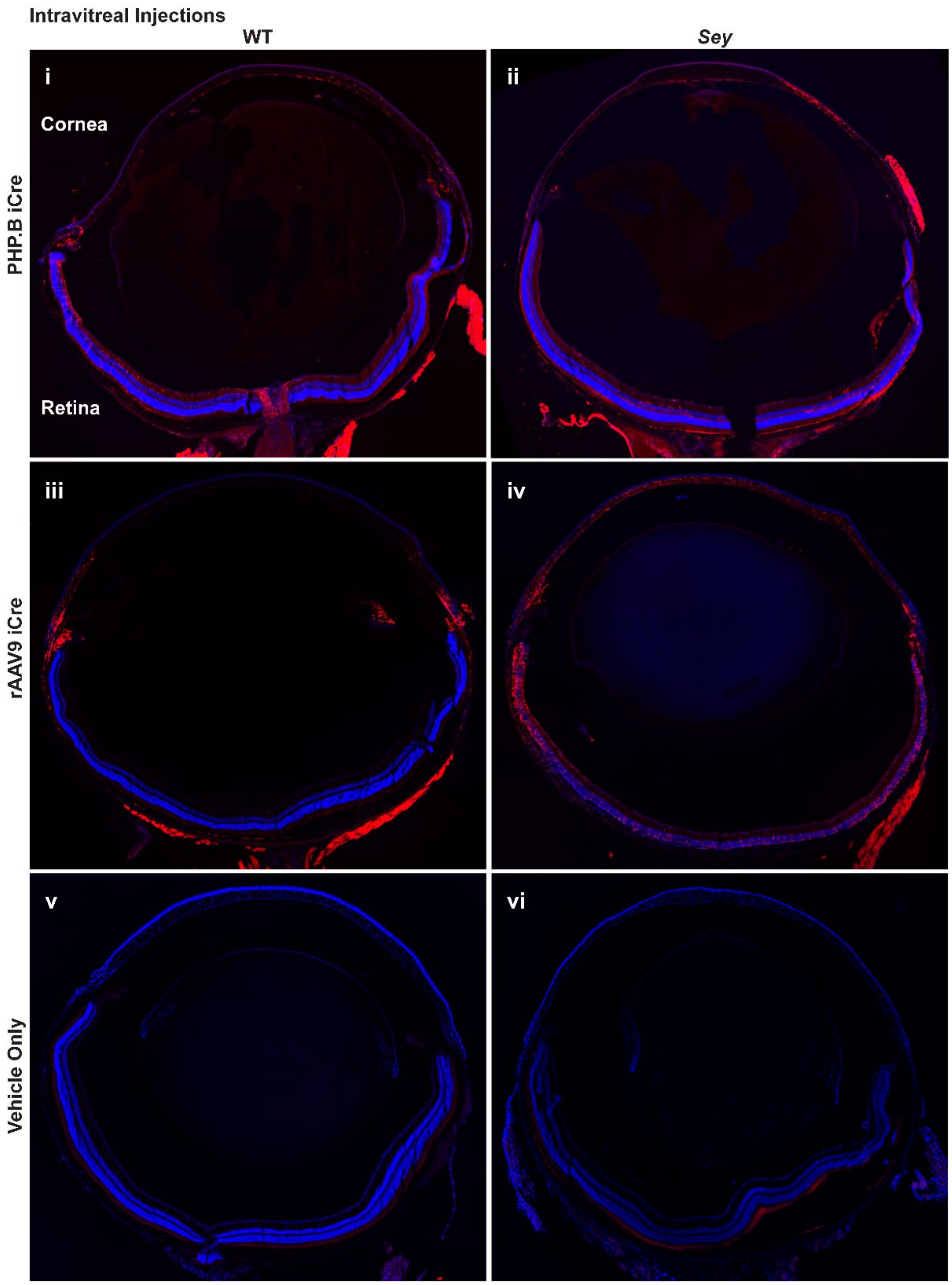

Figure S1. **A variety of retinal and corneal cells were successfully transduced in WT and *Sey* mice after intravitreal delivery of PHP.B and rAAV9 iCre viruses.** Representative overview images of mouse eyes. The Roman numerals correspond to images in Figure 5. Blue, Hoechst nuclear stain; iCre, improved Cre; red, tdTomato epifluorescence; *Sey*, *Pax6* small eye; WT, wild type.

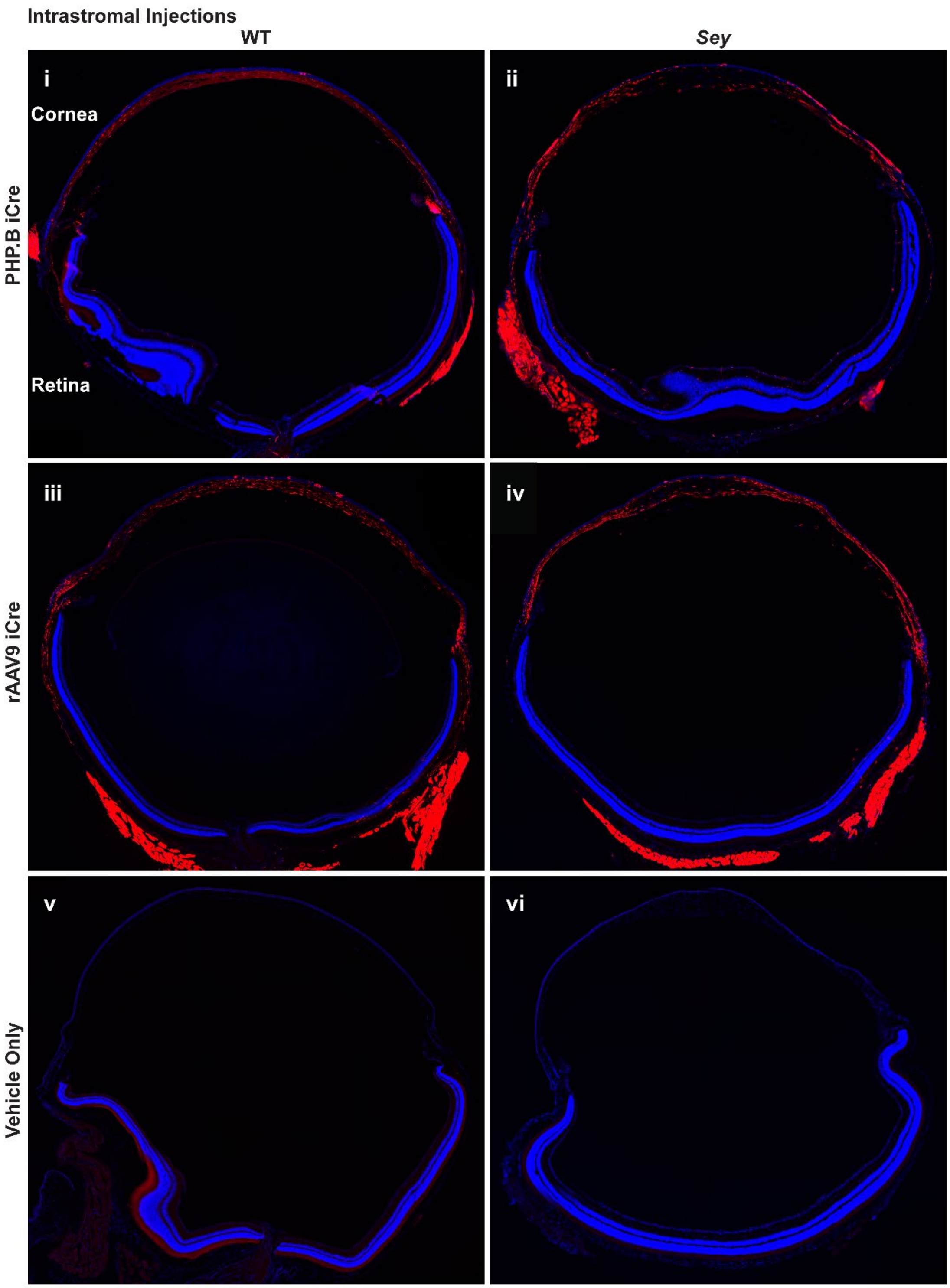

Figure S2. **All three corneal cell layers were successfully transduced in WT and *Sey* mice after intrastromal delivery of PHP.B and rAAV9 iCre viruses.** Representative overview images of mouse eyes. The Roman numerals correspond to images in Figure 6. Blue, Hoechst nuclear stain; iCre, improved Cre; red, tdTomato epifluorescence; *Sey*, *Pax6* small eye; WT, wild type.
